# First episode psychosis: the depressive symptoms and suicidal behaviour that follow

**DOI:** 10.1192/j.eurpsy.2023.2224

**Published:** 2023-07-19

**Authors:** B. L. B. Mesquita, F. Ribeirinho Soares, M. Fraga, M. Albuquerque, J. Facucho, P. Espada, S. Paulino, P. Cintra

**Affiliations:** Department of Psychiatry, Hospital de Cascais, Lisbon, Portugal

## Abstract

**Introduction:**

Depressive symptoms and suicidal behaviour are common among patients that suffered a first-episode psychosis. Depressive symptoms could occur in different phases of psychosis, including in post-psychotic period. Depression is a well-known risk factor for suicidal behaviour in psychosis with data showing that occurrence of depression in psychosis have a significant correlation with suicide risk.

**Objectives:**

The purpose of this paper is todo a brief review on the relation of causality that existes between first episode psychosis and depressive symptoms as well as suicidal ideation.

**Methods:**

Brief non-systematic literature review on the topic.

**Results:**

First episode psychosis is not uncommonly followed by depressive symptoms and suicidal thoughts. The rate of suicide attempt in psychotic patients range from 10 to 50%. Individuals with first episode psychosis have a greater risk of suicidal behavior compared with normal population and chronic disorders. In several studies, factors identified as being associated with depressive symptoms after first episode psychosis were anomalies of psychosocial development, poor premorbid childhood adjustment, greater level of continuing positive symptoms and longer duration of untreated psychosis. Suicidal behavior was associated with sexual abuse, previous suicide attempt, comorbid polysubstance use, lower baseline functioning, longer time in treatment, recent negative events, older patients, longer duration of untreated positive and negative psychotic symptoms, family history of severe mental disorder, depressive symptoms and cannabis use. Data also indicate that treatment and early intervention programs reduce depressive symptoms and suicidal behavior after first episode psychosis.

**Conclusions:**

There is convincing evidence that depressive symptoms and suicidal behaviour have high rates after first episode psychosis. The research for treatment of depressive symptoms and/or suicidal behaviour after first-episode psychosis is very limited, therefore this paper aims to bring to light the importance of more studies on the matter.

**Disclosure of Interest:**

None Declared

